# Child psychiatry: A scientometric analysis 1980-2016

**DOI:** 10.12688/f1000research.12069.1

**Published:** 2017-08-01

**Authors:** Sadiq Naveed, Ahmed Waqas, Salman Majeed, Muhammad Zeshan, Nusrat Jahan, Muhammad Haaris Sheikh

**Affiliations:** 1 KVC Prairie Ridge Psychiatric Hospital, Kansas City, KS, USA; 2 CMH Lahore Medical College & Institute of Dentistry, Lahore Cantt, Pakistan; 3 Pennsylvania State Milton S. Hershey Medical Center, Harrisburg, PA, USA; 4 Bronx-Lebanon Hospital Center, South Bronx, NY, USA; 5 King Edward Medical University, Lahore, Pakistan

**Keywords:** evolution, scientometrics, bibliometric, citespace, child psychiatry, influential, publication output

## Abstract

**Background**: The field of child and adolescent psychiatry lags behind adult psychiatry significantly. In recent years, it has witnessed a significant increase in the publication of journals and articles. This study provides a detailed bibliometric analysis of articles published from 1980 to 2016, in the top seven journals of child and adolescent psychiatry.

**Methods**: Using the Web of Science core collection, we selected 9,719 research papers published in seven psychiatric journals from 1980 to 2016. We utilized the Web of Science Analytics tool and Network Analysis Interface for Literature Studies (NAILS) Project scripts to delineate the general trends of publication in these journals. Then, co-citation analysis and hierarchical cluster analysis was performed using CiteSpace to map important papers, landmark theories and foci of research in child and adolescent psychiatry.

**Results**: The field of child and adolescent psychiatry has experienced an increasing trend in research, which was reflected in the results of this study. Hierarchical cluster analysis revealed that the research foci in psychiatry were primarily studies related to the design of psychometric instruments, checklists, taxonomy, attention deficit hyperactivity disorder (ADHD), depression, PTSD, social phobia, and psychopharmacology. Moreover, several landmark studies, including the validation of a child behavior checklist, Ainsworth's empirical evidence of Bowlby's attachment theory, and adult outcomes of childhood dysregulation were published. This study also reports rapid expansion and innovation in research areas in the field of child and adolescent psychiatry from 1980-2016.

**Conclusions**: Rapid expansion and innovation in research areas in the field of child and adolescent psychiatry has been observed, from 1980 to 2016.

## Introduction

Mental health disorders are very prevalent among children and adolescents, resulting in a significant impact on society. It is estimated that 13–20% of children living in the United States (1 out of 5 children) suffer from a mental disorder every year, resulting in an annual economic loss of 247 billion USD ^1^. Despite these statistics, the field of child psychiatry has attracted a little research interest as compared to other specialties of medicine. This is evident from the fact that not even a single article related to mental health among children was published in the first 45 years of the publication history of American Journal of Insanity ^2^. In a clinical context, the first ever hospital specializing in the treatment of sick children; La’Hôpital des Enfants-Malades, was established in 1802 on the Rue deSévres in Paris. It was also the first time that the field of pediatrics was recognized as an established specialty of medicine ^2^. Institutions specializing in mental health of children, however, did not develop until after World War I, when August Hamberger established his outpatient clinic in the University of Heidelberg ^3^. Experts believe that child psychiatry evolved as a separate field after America’s first juvenile court was established in 1899 ^4^. Then, during World War II, Great Britain started to patronize the psychological development of its children, for a better future ^5^. Similar strides were made in the US, when President Harry Truman declared war on mental illness in 1946, after signing the National Mental Health Act that led to the birth of the National Institute of Mental Health ^6^. This resulted in an explosion of research in understanding the nature of psychiatric diseases, its diagnoses and taxonomy and psychopharmacological and behavioral treatments. At present, child and adolescent psychiatry has established itself as a distinct specialty globally, however, major disparities exist between the developed and third world countries ^7^.

While the development of infrastructure and facilities in any field of medicine are important, scientific research, new discoveries and influential publications are the true marker that ensure its constant progress and evolution. The history of research and development in child psychiatry is quite intricate; spanning discoveries in several domains of medical and social sciences. It is very important to map the research output in a field, to help guide policy makers, researchers and funding agencies towards areas where restriction or increase in research activity is required. In recognition of its importance, several reproducible statistical methods were developed under the umbrella of scientometrics. It is the “quantitative study of science, communication in science, and science policy”, helping to evaluate the impact of journals, scientists and institutes on the development and innovation of a scientific field ^8^.

The present study analyzes the trends of research in the field of child and adolescent psychiatry by employing reproducible scientometric techniques. Although several scientometric studies have been published in general psychiatry ^9, 10^ and other fields of medicine, there is a paucity of such studies mapping the research output in the field of child psychiatry, hence warranting this study. The present study identifies influential publications, landmark theories, authors, countries and major funding agencies contributing to child psychiatry from 1980–2016.

## Methods

For the purpose of this scientometric analysis, we selected seven journals ( Table 1) indexed under the term “Child Psychiatry” in Google Scholar. These journals were selected on the base of their ISI impact factor, h5-index and h5-median, following the methodology of previous scientometric articles published in the field of psychiatry ^9, 10^. The Web of Science core collection was utilized to download bibliographic records of articles published in these journals from 1980–2016 to provide an overview of recent advances in this field. These records included title, author names, abstract, key words and cited references. This search was performed in December, 2016 and records for a total of 9,719 articles, published during 1980- June 2016 were retrieved. There were no restrictions based on type or language of articles included in this analyses.

**Table 1.** Impact factor of included journals (n=7).

Journal	H5-index	H5-Median	Impact factor
Journal of Child Psychology and Psychiatry	67	101	6.615
Journal of the American Academy of Child & Adolescent Psychiatry	67	97	7.182
European Child & Adolescent Psychiatry	41	67	3.339
Child Psychiatry & Human Development	26	45	1.839
Child and Adolescent Psychiatric Clinics of North America	24	32	1.590
Clinical Child Psychology and Psychiatry	21	28	1.192
Child and Adolescent Psychiatry and Mental Health	20	31	2.134

This study utilized three software tools for analyses of data; Web of Science core collection records, Network Analysis Interface for Literature Studies (NAILS) Project scripts ^11^ and Citespace (v4.0 R5, Drexel University, Pennsylvania, USA) ^12^. The Web of Science core collection-online analysis platform was used to document journal wise influential authors, institutions, funding agencies and countries. NAILS software was utilized to analyze these to identify the most cited keywords in these journals.

CiteSpace (v4.0 R5, Drexel University, Pennsylvania, USA) is a Java-based platform that allows knowledge mapping by visualization of bibliographic data and hence it is a popular and user-friendly tool to perform co-citation analyses ^12^. According to the theory of document co-citation ^13, 14^, co-citation relationships between two documents exist when they are cited together by another document.

Using “time slicing”, the bibliographic records were divided into four groups according to the year of publication; 1980–1990, 1991–2000, 2001–2010 and 2011–2016, the year per slice was set to 1 with each year represented by top 50 articles based on the number of citations. The ‘team source’ selects were ‘title’, ‘abstract’, ‘author keywords’ and ‘keywords plus’ and the ‘node types’ selected were ‘cited reference’.

Network analyses were run with the link reduction method using pathfinder network scaling and then, bibliographic records for each 10-year slice were visualized separately. Articles were represented as nodes and lines as edges. Using this technique, several key results could be identified, such as new theories/concepts related to a field (visualized as a purple ring), centrality reflecting the status of a publication in their network/field, citation bursts (hot topics of research), citation tree rings representing year-wise citation pattern of a node (article). Articles with centrality values > 0.1 were considered as significant entities controlling significant resources in their collaborative networks. Based on these techniques, researchers can observe and understand bibliographic trends in order to identify patterns of research in particular fields and regularities of citations in particular time periods ^15^.

## Results

### General trends of publication and citation output

A total of 9, 719 research papers were published in the seven psychiatric journals ‘Journal of Child Psychology and Psychiatry’, ‘Journal of the American Academy of Child & Adolescent Psychiatry’, ‘European Child & Adolescent Psychiatry’, ‘Child Psychiatry & Human Development’, ‘Child and Adolescent Psychiatric Clinics of North America’, ‘Clinical Child Psychology and Psychiatry’, and ‘Child and Adolescent Psychiatry and Mental Health’ ( Table 1), from 1980 to June 2016. All journals publish multidisciplinary research articles in the fields of child and adolescent psychiatry, ensuring constant improvement and evolution of the field toward a cutting-edge, evidence-based clinical specialty.

**Figure 1.**
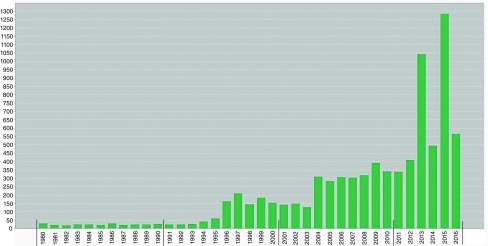
Publication output of child psychiatry journals included in present analyses, from 1980–2016.

**Figure 2.**
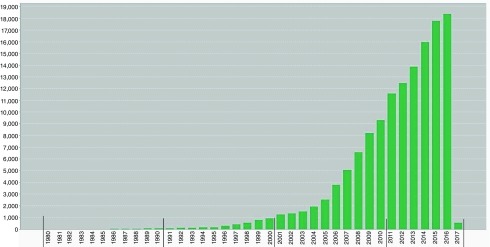
Trend of number of citations received by research articles published in the top seven journals of child and adolescent psychiatry from 1980–2016.

Figure 1 shows the yearly publication volume in the field of child and adolescent psychiatry since 1980, and documents the rapid increase in publication volume in this field since the 1980s. The publication output in these journals rose from less than a 100 journal articles in 1980 to more than 500 in 2015. Figure 2 shows the yearly trend in the number of citations received by articles included in our analyses. The articles were cited a total of 1,37,006 times, however, this number dropped to 1,27,119 after removing self-citations. The total number of articles citing these publications was 81551 (77853 after excluding self-citations). Average citations per item were 16.85, contributing to an h-index of 132.

The increasing trend in publication ensures that the field of child psychiatry is constantly evolving because of new discoveries in epidemiology, assessment techniques, genetics, neurosciences and therapeutics. However, the research output in journals related to child psychiatry still lags behind those of general psychiatry and other specialties of medicine ^16^.

### Top institutions, authors and countries

It is interesting to note that the highest output of research in child psychiatry comes from institutions in developed countries. According to Albayrak *et al.*, this regional disparity is attributed to a high GDP of these countries, higher funding available and availability of more public health resources, specialty training programs and mental health professionals committed to the field of child psychiatry ^7^. In addition, regions like South Asia, and a high percentage of developing European countries (23%) do not have training programs in child psychiatry, hence, low research output ^7^. Similar trends were identified in our study. According to the Web of Science (core database) citation report for these seven journals, countries with highest research output were USA, England, Netherlands, Germany, Spain, Canada, Australia, Sweden, Switzerland and Norway. Globally, the most productive organizations were University of London, Kings College London, Yale University, University of California, University College, London, Harvard University, Pensylvania Commonwealth System of Higher Education, Vrije Universiteit Amsterdam, Radboud University Nijmegen and the University of Pittsburgh in USA ( Table 2).

**Table 2.** Top organizations, countries and authors from 1980–2016 (n= 8,131).

Most Productive author	n	Country	n	Organization	n
Buitelaar JK	113	USA	3216	University of London	698
Rothenberger A	78	England	1337	Kings College London	478
Verhulst FC	76	Netherlands	651	Yale University	290
Sonuga-Barke EJS	70	Germany	567	University College London	287
Coghill D	66	Spain	375	University of California	267
Banaschewski T	62	Canada	353	Harvard University	247
Steinhuasen HC	59	Australia	312	Pensylvania Commonhealth System of Higher Education	199
Gillberg C	48	Sweden	257	Vrije Universiteit Amsterdam	151
Rutter M	48	Switzerland	214	Radboud University Nijmegen	148
Butter M	48	Norway	206	University of Pittsburgh	140

### Most cited keywords from 1980 to 2016

**Figure 3.**
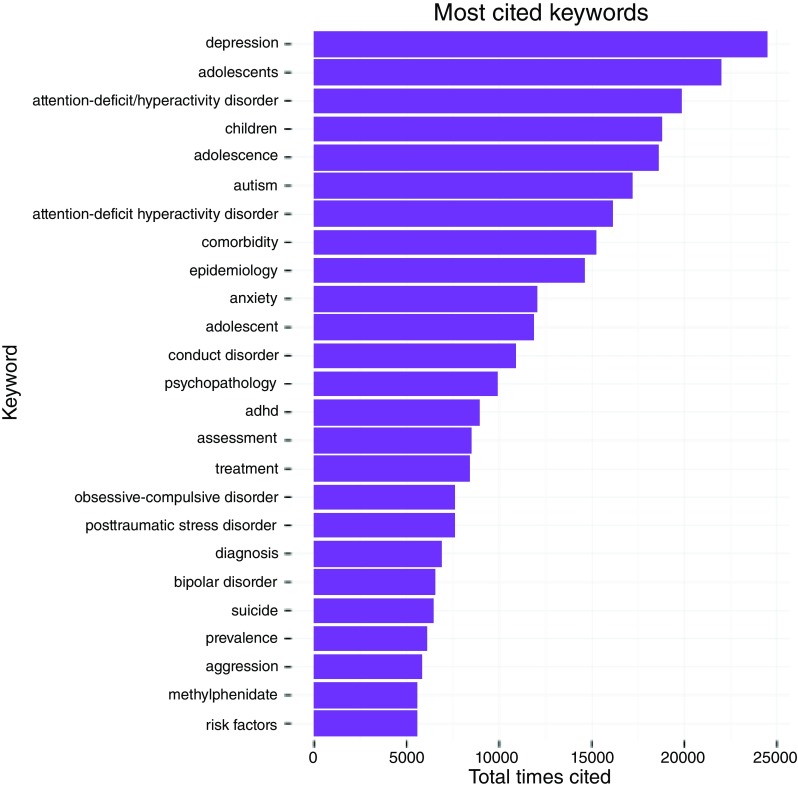
Most cited keywords in the research articles published in journals included in the present analysis.

According to our analysis, the top foci of research in child psychiatry correspond with most common mental health conditions globally. Figure 3 details the top cited keywords, representing the top foci of research in these selected journals ( Figure 3). Top cited psychopathologies were depression, attention deficit hyperactivity disorder (ADHD), autism, anxiety, conduct disorder, obsessive compulsive disorder, post-traumatic stress disorder, bipolar disorder, suicide and aggression. This is in accordance with Polanzcyk *et al.,* who identified the worldwide prevalence of any anxiety disorder to be 6.5%, any depressive disorder to be 2.6%, ADHD to be 3.4%, and any disruptive disorder to be 5.7% ^17^. Methylphenidate was identified as the top cited keyword for a drug used in child psychiatry. Our results are in accordance with López Muñoz *et al.*, who reported methylphenidate to be the most researched drug for attention deficit hyperactivity disorder (ADHD), also correlating it with an increased trend in its use ^18^.

### Publications from 1980 to 1990

**Figure 4.**
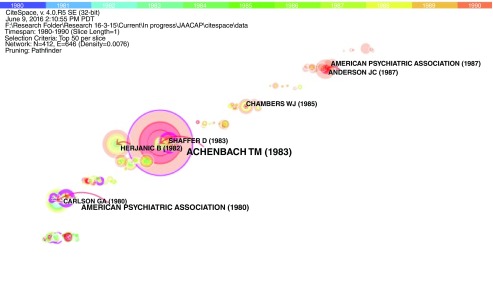
Visualization of important nodes from 1980–1990. Figure 4 represents a visual co-citation network of 412 research documents published in the field of child psychiatry from 1980–1990. The rings represent several key results such as new theories/concepts related to a field (visualized as a purple ring), centrality reflecting the status of a publication in their network/field, and citation tree rings representing year-wise citation pattern of an article.

In further analysis, CiteSpace was used to identify important articles based on their centrality values. Articles with centrality values > 0.1 were considered significant. These articles were considered important within their collaborative network, focused on a specific research domain ( Table 3, Figure 4). Visualization of these clusters also helped in identification of purple nodes that represent important and groundbreaking theories, and represent a link between two different clusters ^12^.

**Table 3.** Top articles with centrality values ≥ 0.1 or purple rings (1980–1990).

Reference	Centrality value	Title	Purple node
Carlson and Cantwel (1980)	0.42	Unmasking masked depression in children and adolescents	Yes
Shaffer D *et al*. (1983)	0.31	A children's global assessment scale (CGAS)	Yes
Carroll BJ (1980)	0.24	Testing communicative performance: An interim study	No
Gaensbauer and Sands (1979)	0.23	Distorted affective communications in abused/neglected infants and their potential impact on caretakers	Yes
Achenbach (1980)	0.22	DSM-III in light of empirical research on the classification of child psychopathology.	Yes
Egeland and Sroufe (1981)	0.21	Attachment and early maltreatment	Yes
Ainsworth *et al* (1978)	0.18	Patterns of attachment: A psychological study of the strange situation	Yes
Cantwell (1978)	0.15	Hyperactivity and antisocial behavior.	Yes
Achenbach (1983)	0.12	Manual for the child behavior checklist and revised child behavior profile.	Yes
Lewis *et al* (1979)	0.1	Violent juvenile delinquents: Psychiatric, neurological, psychological, and abuse factors	Yes

From 1980–1990, there were 412 nodes (articles) and 646 edges (co-citation links). The most important paper with a centrality value of 0.42 (identified as a purple node) was titled, “diagnostic significance of masked depression”, by Carlson & Cantwell ^19^. It was a landmark study in the field of child psychiatry as it elucidated the diagnostic significance of unmasking depression in adolescents who presented with other comorbidities and symptoms. Cantwell reviewed the evidence linking hyperactivity with antisocial behavior in the youth,and Lewis *et al.* compared the neuropsychiatric, intellectual, and educational status of extremely violent and less violent incarcerated boys ^20, 21^.

Development of questionnaires and rating scales specific to child psychiatry is another milestone in its history. Like any other scientific discipline, this allowed researchers and clinicians to reliably quantify emotional problems among children and adolescents. This also helps them to track symptoms and response to the treatment ^22^. During this decade, two important rating scales were developed, subsequently influencing research in child psychiatry. Shaffer *et al.’*s ‘Children’s Global Assessment Scale’ adapted the adult version of Global Assessment Scale, to evaluate overall functioning among children, as a complement to their clinical diagnoses ^23^. Similarly, Achenbach ’s exploration of DSM-III in perspective of child psychopathology lead to the development Child Behavior Checklist that integrates information from a variety of sources; parent, child and teacher ^24, 25^.

Other important publications explored the relationship between the caretakers and the child. Bowlby’s attachment theory propounded that a child initially forms only one attachment relationship, and this attachment figure becomes a base for all future relationships ^26^ and disrupting this, can lead to long term consequences. Ainsworth’s studies provided the first empirical evidence of Bowlby’s attachment theory ^26^, which was subsequently explored in a longitudinal study by Egeland and Sroufe ^27^. In a similar context, Gaensbauer & Sands’ study on the therapeutic relationship between abused/neglected infants and their caretakers, which concluded that personality traits of the child may contribute to disturbance in caretaker-infant interaction, leading to abuse and neglect ^28^. All of these studies represented significant between-ness centrality in this time period.

### Publications from 1991 to 2000

**Figure 5.**
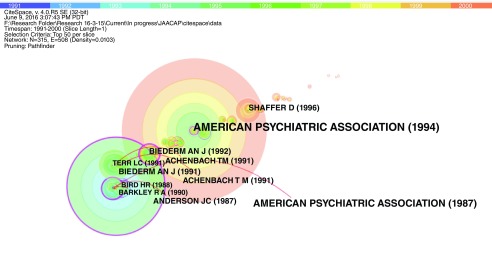
Visualization of important nodes from 1991–2000. Figure 5 represents a visual co-citation network of 315 research documents published in the field of child psychiatry from 1991–2000. The rings represent several key results such as new theories/concepts related to a field (visualized as a purple ring), centrality reflecting the status of a publication in their network/field, and citation tree rings representing year-wise citation pattern of an article.

There were 315 nodes and 508 edges in this time period ( Table 4, Figure 5). In recent decades, the integration of epidemiological evidence into child psychiatry, has truly helped it reach its scientific potential. This specific discipline helped child psychiatry in three principal ways: a) identify the burden of childhood psychiatric illnesses b) identify new risk factors for psychiatric illnesses and c) explore the validity and reliability of diagnostic statistical manual.

**Table 4.** Top articles with centrality values ≥ 0.1 or purple rings 1991–2000.

Reference	Centrality value	Basic theme	Purple node
Brent *et al*. (1988)	0.34	Risk factors for adolescent suicide: a comparison of adolescent suicide victims with suicidal inpatients	Yes
Anderson *et al*. (1987)	0.31	DSM-III disorders in preadolescent children: Prevalence in a large sample from the general population	Yes
Biederman *et al*. (1992)	0.26	Further evidence for family-genetic risk factors in attention deficit hyperactivity disorder: Patterns of comorbidity in probands and relatives in psychiatrically and pediatric ally referred samples.	Yes
American Psychiatric Association (1987)	0.25	Committee on nomenclature and statistics. Diagnostic and Statistical Manual of Mental Disorders, Revised Third Edition.	Yes
Bird *et al*. (1988)	0.19	Estimates of the prevalence of childhood maladjustment in a community survey in Puerto Rico: The use of combined measures	Yes
Barkley *et al*. (1990)	0.17	The adolescent outcome of hyperactive children diagnosed by research criteria: I. An 8-year prospective follow-up study	Yes
Lewinsohn *et al*. (1993)	0.13	Adolescent psychopathology: I. Prevalence and incidence of depression and other DSM-III—R disorders in high school students	Yes
Schwab-Stone *et al*, (1994)	0.12	Reliability of diagnostic reporting for children aged 6-11 years: a test- retest study of the Diagnostic Interview Schedule for Children-Revised	No
Bird *et al*. (1993)	0.12	Patterns of diagnostic comorbidity in a community sample of children aged 9 through 16 years.	No
Cohen *et al*. (1993)	0.11	An epidemiological study of disorders in late childhood and adolescence—I. Age‐and gender‐specific prevalence	Yes
American Psychiatric Association (1994)	0.1	Diagnostic and statistical manual of mental disorders (DSM)	No
Pynoos *et al.* (1987)	0.1	Life threat and posttraumatic stress in school age children.	No
Lahey *et al.* (1994)	0.1	DSM-IV field trials for oppositional defiant disorder and conduct disorder in children and adolescents	Yes

During this period, several landmark epidemiological studies were conducted. There were three studies focusing on suicide, PTSD and ADHD. The most important paper with a centrality value of 0.34, identified as a purple node was entitled, “Risk factors for adolescent suicide: a comparison of adolescent suicide victims with suicidal inpatients” by Brent *et al.* ^29^. It identified the most prevalent risk factors for suicidal behaviors in adolescents and emphasized on their proper identification. Pynoos *et al.*’s work on acute PTSD garnered a lot of attention during this period. He concluded that these symptoms were not affected by age, gender and ethnicity and that severity of acute PTSD symptoms correlated with proximity to violence ^30^. Barkley *et al*. identified hyperactivity as a pattern of behavioral symptom that is highly stable over time and associated with considerably greater risk for family disturbance and negative academic and social outcomes in adolescence ^31^.

Although DSM-III was published in 1980, it did not appear as an influential entity in child psychiatry during 1980s. However, it attracted a lot epidemiological studies from 1990–2000, mainly because it operationalized the diagnostic criteria of mental illnesses and used a phenomenological approach. Bird *et al*. (1993) in his epidemiologic study identified patterns of the comorbidity of four major diagnostic domains (attention deficit disorders, conduct/oppositional disorders, depression and anxiety disorders) among children ^32^, and Cohen *et al*. (1993) reported that patterns of diagnoses varied by both age and gender ^33^. Subsequent publications include Anderson *et al.*’s work investigating the prevalence of DSM-III disorders in preadolescent children ^34^, where it was found that the most prevalent disorders were attention deficit, oppositional and separation anxiety disorders. And the least prevalent were depression and social phobia. Bird *et al.* delineated the demographic correlates of maladjustments and its DSM-III diagnostic domains ^35^.

The publication of a DSM III- revised version by the American Psychiatric Association was significant in their own collaborative network, as well as visualized as a purple node representing a landmark work in the field of child psychiaty ^36^. Using DSM III- R critera, Lewinsohn *et al.* identified the prevalence and incidence of depression ^37^ and other DSM-III-R disorders among high school students. This decade also included the development of Diagnostic Interview Schedule for Children-Revised (DISC-R), to be used among children, by Schwab-Stone *et al*. ^38^. This was a very important development, as it could be administered by clinicians as well as lay interviewers with no formal clinical training ^38^.

The decade also included DSM- IV publication by American Psychiatric Association as an important publication ^39^. A study by Lahey *et al.* compared the psychometric properties of DSM- IV criteria for oppositional defiant disorder and conduct disorder with previous DSM diagnostic formulations ^40^. It concluded that DSM-IV definitions and validity of oppositional defiant disorder and conduct disorder are somewhat better than DSM-III-R definitions.

The introduction of cutting edge techniques in child psychiatry integrated new disciplines such as genetics. In 1992, Biederman *et al.* provided the evidence for family-genetic influences in the development of ADHD ^41^. Table 5 provides a detailed analysis of the articles selected based on their centrality values. Please, note that some of these articles were published in the previous decade but they influenced other research in this decade.

**Table 5.** Top articles with centrality values ≥ 0.1 or purple rings (2001–2010).

Reference	Centrality value	Basic theme	Purple node
March *et al*. (2004)	0.26	Treatment for Adolescents With Depression Study (TADS) Team: Fluoxetine, cognitive-behavioral therapy, and their combination for adolescents with depression: Treatment for Adolescents With Depression Study (TADS) randomized controlled trial	Yes
Shaffer *et al*. (2000)	0.24	NIMH Diagnostic Interview Schedule for Children Version IV (NIMH DISC- IV): description, differences from previous versions, and reliability of some common diagnoses.	Yes
Kim-Cohen *et al*. (2003)	0.18	Prior juvenile diagnoses in adults with mental disorder: developmental follow- back of a prospective-longitudinal cohort.	Yes
Angold *et al*. (1998)	0.18	Perceived parental burden and service use for child and adolescent psychiatric disorders	Yes
Ford *et al*. (2003)	0.17	The British child and adolescent mental health survey	Yes
Shaffer *et al*. (1996)	0.16	The NIMH Diagnostic Interview Schedule for Children Version 2.3 (DISC-2.3): Description, acceptability, prevalence rates, and performance in the MECA study.	Yes
Faraone *et al*. (2005)	0.16	Molecular genetics of attention-deficit/hyperactivity disorder	Yes
Angold *et al*. (1999)	0.15	Comorbidity	
Castellanos *et al*. (2002)	0.15	Developmental trajectories of brain volume abnormalities in children and adolescents with attention-deficit/hyperactivity disorder	Yes
Leibenluft *et al*. (2003)	0.14	Defining clinical phenotypes of juvenile mania.	Yes
Costello *et al*. (1996)	0.14	The Great Smoky Mountains Study of Youth: goals, design, methods, and the prevalence of DSM-III-R disorders	Yes
Gottesman *et al*. (2003)	0.13	The endophenotype concept in psychiatry: etymology and strategic intentions.	Yes
MTA Cooperative Group (1999)	0.12	A 14-month randomized clinical trial of treatment strategies for attention- deficit/hyperactivity disorder	Yes
Conners (1997)	0.12	Conners' Rating Scales--revised: User's Manual	Yes
American Psychiatric Association (2000)	0.11	Diagnostic criteria from DSM-IV-tr	Yes
Goodman *et al*. (2000)	0.11	Using the Strengths and Difficulties Questionnaire (SDQ) to screen for child psychiatric disorders in a community sample.	No
Costello *et al*. (2005)	0.11	10-year research update review: the epidemiology of child and adolescent psychiatric disorders: I. Methods and public health burden	Yes
Walkup *et al*. (2001)	0.1	Fluvoxamine for the treatment of anxiety disorders in children and adolescents.	Yes
MTA Cooperative Group (1999)	0.1	Moderators and mediators of treatment response for children with attention- deficit/hyperactivity disorder: the Multimodal Treatment Study of children with Attention-deficit/hyperactivity disorder	Yes
Pine *et al*. (1998)	0.1	The risk for early-adulthood anxiety and depressive disorders in adolescents with anxiety and depressive disorders	Yes

### Publications from 2001 to 2010

**Figure 6.**
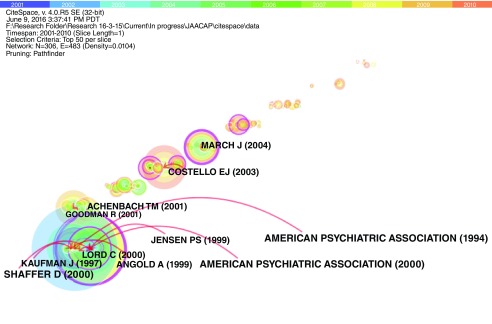
Visualization of important nodes from 2001–2010. Figure 6 represents a visual co-citation network of 306 research documents published in the field of child psychiatry from 2001–2010. The rings represent several key results such as new theories/concepts related to a field (visualized as a purple ring), centrality reflecting the status of a publication in their network/field, and citation tree rings representing year-wise citation pattern of an article.

There were 306 nodes and 483 edges ( Table 5, Figure 6). An eminent scientist in the field of child psychiatry, Angold *et al*. (1999) conducted a meta-analysis to provide an understanding of comorbidity of different psychiatric disorders ^42^. Angold *et al*. Also highlighted that severity of symptomatology among children and the resulting impairment contributed significantly to parents’ burden ^43^. Costello *et al*. conducted a 10 year review update to track the recent progress in child and adolescent psychiatric epidemiology ^44^. It summarized the burden and available methods to screen and diagnose mental illnesses among the youth ^44^. Similarly, Ford *et al.* identified the prevalence of DSM-IV disorders by conducting the child and adolescent mental health survey ^45^.

This decade experienced an influence from a number of landmark prospective cohort studies. Prospective cohort studies provide strong evidence regarding temporality and causality, and minimize recall bias. The studies revolutionized the understanding of the developmental course of psychopathologies. Kim-Cohen *et al*.’s work (2003) emphasized the public health importance of juvenile disorders by concluding that most adult disorders might be an extension of juvenile disorders ^46^. Pine *et al*. identified anxiety and depressive disorders in adolescence to be a strong risk factor for early-adulthood anxiety and depressive disorders ^47^. Costello *et al*. (1996) influenced this era by their renowned “The Great Smoky Mountain study”, which looked at the prevalence of psychiatric disorders in urban and rural population ^48^. The study identified several key findings, such as a high burden of psychiatric illnesses among rural populations, externalizing and internalizing dimensions of psychopathologies, and poverty and puberty as a risk factor for depression ^48^.

In 2000, the American Psychiatric Association published the DSM-IV-TR ^49^. Similar to its previous versions, DSM-IV-TR brought about landmark changes (visualized as a purple node) in the field of child psychiatry

In this decade, a particular emphasis was also observed in the study of genetic –environment interaction in development and progression of diseases. These studies stimulated a great deal of research in the domain of psychiatric genetics especially in ADHD and mania. Castellanos *et al*. concluded that genetic and/or early environmental influences on brain development in ADHD are fixed, non-progressive, and unrelated to stimulant treatment ^50^. Faraone *et al*. reviewed important genetic variances and its association with ADHD ^51^. Leibenluft *et al*. described the clinical phenotypes of juvenile mania ^52^. Gottesman and Gould’s work emphasized the importance of endo-phenotypes in the understanding of neurobiological correlates of psychiatric disorders ^53^

Similar to previous decades, the work in this period also focused on the importance of assessment, diagnoses and taxonomy of childhood psychiatric disorders. Two scales in particular provided a strong base for screening, diagnoses and research pertaining to ADHD. The Conners' Rating Scales-Revised User Manual published in 1997 garnered a lot of attention in this decade ^54^. The scale represents validated instrument with excellent psychometric properties for evaluation, diagnosis, and treatment response of children with ADHD and co-morbid disorders ^54^. Goodman *et al.* employed the Strengths and Difficulties Questionnaire (SDQ) to screen child psychiatric disorders in a community sample ^55^. Owing to their inexpensive use by non-trained individuals, these scales are extensively used in screening ADHD among children in school settings, community settings as well as research.

Providing an updated version of DISC-R, Shaffer *et al* assessed the reliability of NIMH Diagnostic Interview Schedule for Children Version 2.3 (DISC-2.3) in the MECA study ^56^. Subsequent papers included Shaffer *et al*.’s comparison of NIMH Diagnostic Interview Schedule for Children Version IV (NIMH DISC-IV) with its previous versions, and its reliability for some common diagnoses ^57^.

In this decade, a lot of pharmacological research was guided by several influential studies on depression, anxiety and ADHD among children. The most important paper with a centrality value of 0.26 and identified as a purple node was titled “Treatment for adolescents with depression Study (TADS) Team: Fluoxetine, cognitive-behavioral therapy, and their combination for adolescents with depression” by March *et al*. ^58^. This study concluded that the combination of CBT and SSRI is the most efficacious for treating major depression among adolescent population. It also helped guide the National Institute for Health and Clinical Excellence (NICE) guidelines for treating adolescent depression.

Another trial proved that fluvoxamine is efficacious in childhood and adolescent anxiety disorders ^59^. The MTA Cooperative Group tested the treatment strategies for ADHD ^60^ and also identified the moderators and mediators of treatment response for children with ADHD ^61^. This was one of the most influential study guiding future research on treatment of ADHD. Since 1999, when the NIMH study was published, thousands of additional peer-reviewed studies have been published on the topic of ADHD treatment.

### Publications from 2011 to 2016

**Figure 7.**
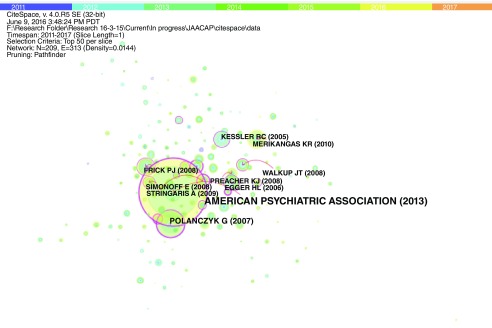
Visualization of important nodes from 2011- July 2016. Figure 7 represents a visual co-citation network of 209 research documents published in the field of child psychiatry from 2011–2016. The rings represent several key results such as new theories/concepts related to a field (visualized as a purple ring), centrality reflecting the status of a publication in their network/field, and citation tree rings representing year-wise citation pattern of an article.

There were 209 nodes and 313 edges ( Table 6, Figure 7). “The fifth edition of Diagnostic and statistical manual of mental disorders (DSM-5)” was identified as the most important publication. ^62^. Willcutt *et al*. ^63^ reviewed the validity of DSM-IV attention deficit/hyperactivity disorder symptom dimensions and subtypes. Our analysis identified two important works in epidemiology; elucidating the prevalence of childhood psychiatric disorders. In his meta-analysis, Polanczyk analyzed the causes for worldwide variation in estimates of ADHD ^64^. Kessler *et al*. ^65^ conducted a survey to estimate the lifetime prevalence and age of onset of distributions of DSM-IV disorders in the National Comorbidity Survey Replication.

**Table 6.** Top articles with centrality values ≥ 0.1 or purple rings (2011–2016).

Reference	Centrality value	Basic theme	Purple node
American Psychiatric Association (2013)	0.35	Diagnostic and statistical manual of mental disorders (DSM-5)	Yes
Leibenluft (2011)	0.31	Severe mood dysregulation, irritability, and the diagnostic boundaries of bipolar disorder in youths.	Yes
Polanczyk *et al*. (2007)	0.27	The worldwide prevalence of ADHD: a systematic review and meta- regression analysis.	Yes
Althoff *et al*. (2010)	0.24	Adult outcomes of childhood dysregulation: a 14-year follow-up study.	Yes
Stringaris and Goodman (2009)	0.22	Longitudinal outcome of youth oppositionality: irritable, headstrong, and hurtful behaviors have distinctive predictions	Yes
Sonuga-Barke *et al*. (2013)	0.2	. Nonpharmacological interventions for ADHD: systematic review and meta-analyses of randomized controlled trials of dietary and psychological treatments.	Yes
Nylund *et al*. (2007)	0.16	Deciding on the number of classes in latent class analysis and growth mixture modeling: A Monte Carlo simulation study	Yes
Birmaher *et al*. (2007)	0.16	Practice parameter for the assessment and treatment of children and adolescents with depressive disorders.	Yes
Egger and Angold (2006)	0.15	Common emotional and behavioral disorders in preschool children: presentation, nosology, and epidemiology	Yes
Frick and White (2008)	0.13	Research review: The importance of callous‐unemotional traits for developmental models of aggressive and antisocial behavior.	Yes
Simonoff *et al*. (2008)	0.13	Psychiatric disorders in children with autism spectrum disorders: prevalence, comorbidity, and associated factors in a population-derived sample.	Yes
Walkup *et al*. (2008)	0.13	Cognitive behavioral therapy, sertraline, or a combination in childhood anxiety	Yes
Copeland *et al*. (2009)	0.13	Childhood and adolescent psychiatric disorders as predictors of young adult disorders.	Yes
Kessler *et al*. (2005)	0.12	Lifetime prevalence and age-of-onset distributions of DSM-IV disorders in the National Comorbidity Survey Replication	Yes
Silverman *et al*. (2008)	0.12	Evidence-based psychosocial treatments for phobic and anxiety disorders in children and adolescents.	Yes
Wolke *et al*. (2009)	0.11	Selective drop-out in longitudinal studies and non-biased prediction of behaviour disorders.	Yes
Willcutt *et al*. (2012)	0.11	Validity of DSM-IV attention deficit/hyperactivity disorder symptom dimensions and subtypes.	Yes
Stringaris and Goodman (2009)	0.1	Three dimensions of oppositionality in youth	Yes

This decade also attracted a lot of research on disruptive disorders among children. Stringaris & Goodman ^66^ recognized three dimensions of oppositionality in youth; irritability, hurtful and headstrong, deeming them as differential predictors of aetiology, prognosis and treatment responsiveness. Later, Stringaris & Goodman assessed the longitudinal outcomes of these three dimensions, with irritability predicting depression and anxiety, headstrong dimension with ADHD and hurtful dimension predicting aggressive conduct disorder ^67^. White ^68^ reviewed the importance of callous traits for developmental models of aggressive and antisocial behavior and Wolke *et al*. ^69^ investigated whether drop out in the Avon Longitudinal Study of Parents And Children (ALSPAC) is systemic or random and if systematic, whether it had an impact on the prediction of disruptive behavior disorders.

Egger & Angold (2006) conducted an important review about common emotional and behavioral disorders in preschool children ^70^. Subsequent papers include Leibenluft’s work on severe mood dysregulation, irritability, and the diagnostic boundaries of bipolar disorder in youth ^71^. It emphasized that severe mood dysregulation disorder is a different diagnostic entity than Bipolar Disorder, while Frick and Simonoff *et al*. ^72^ elucidated that social anxiety disorders, ADHD and conduct disorders were most comorbid with autism spectrum disorders.

Two prospective studies influenced research in this decade. These included Althoff *et al*’s 14-year follow-up study, concluding childhood dysregulation identified using the child behavior checklist-dysregulation profile (CBCL-DP) could predict anxiety and behavior disruptive disorders in adulthood ^73^. Copeland *et al* ^74^ identified childhood and adolescent psychiatric disorders as predictors of young adult disorders.

Walkup *et al*. ^75^ published a randomized controlled trial to assess treatment options of childhood anxiety disorders. They concluded that a combination of cognitive behavioral therapy and Sertraline to be of greatest efficacy in childhood anxiety. And Birmaher & Brent identified practice parameters for the assessment and treatment of children and adolescents with depressive disorders ^76^. This decade also included two influential studies on non-pharmacological interventions. In contrast to previous studies that had focused on pharmacological treatments for ADHD, Sonuga-Barke *et al*. reviewed the evidence for non-pharmacological interventions ^77^ such as Free fatty acid supplementation, artificial food color exclusion, behavioral interventions, neurofeedback, cognitive training, and restricted elimination. Similarly, Silverman *et al*. ^78^ reviewed the evidence-based psychosocial treatments for phobic and anxiety disorders in children and adolescents.

This decade was also particularly influenced by Nylund and colleagues’ work on latent class analysis and mixture modeling techniques, commonly used in behavioral and social science research for identifying patterns of behaviors, psychiatric symptoms and disorders, and co-occurrence of aspects of the social environment ^79^.

Source data utilized in this study, compiled into text documents The data is also accessible via the Clarivate analytics Web of Science Core database. Click here for additional data file. Copyright: © 2017 Naveed S et al. 2017 Data associated with the article are available under the terms of the Creative Commons Zero "No rights reserved" data waiver (CC0 1.0 Public domain dedication).

## Discussion

The field of child psychiatry is still a relatively new and expanding field, in comparison to other specialties of medicine. It has evolved significantly over the last few years. Most of the literature in this field was contributed by United States of America and the European countries, with small contributions from the developing countries. Similar trends were observed in the regional distribution of funding agencies and authors contributing to this field. Several pharmaceutical industries were also identified among top ten funding agencies. A number of landmark papers and research foci were also identified. During the first three decades, the researchers focused on assessment tools, taxonomy, identification of risk factors, and symptomatology. Over time, there has been a growing interest in finding better assessment tools and more effective psychopharmacological options. However, more recently, there has been a rapid development in several areas including neurobiology, neuroimaging, and molecular genetics. Researchers are more interested in exploring etiological factors leading to psychiatric illnesses. The authors strongly believe that these innovative trends in the field would help identify and manage the childhood psychiatric disorders at an earlier stage, and also increase the quality of life among patients, their families and caretakers.

## Data availability 

**Dataset 1: Source data utilized in this study, compiled into text documents.** The data is also accessible via the Clarivate analytics Web of Science Core database. DOI, 10.5256/f1000research.12069.d170670 ^80^
