# Supplementary material for: Child psychiatry: A scientometric analysis 1980-2016
Source: F1000Res. 2017 Aug 1;6:1293. [Version 1] doi: 10.12688/f1000research.12069.1 (PMC5585878; doi:10.12688/f1000research.12069.1)
Supplement: Peer review history

# Peer review: Referee response for version 1

**DOI:** 10.5256/f1000research.13058.r25167

**Published:** 2017-09-05

**Authors:** Muhammad Waqar Azeem

**Affiliations:** 1 Sidra Medical and Research Center, Weill Cornell Medical College in Qatar, Doha, Qatar

Excellent work by authors in reviewing the literature on child and adolescent psychiatry.

 Would be important to mention in the limitations that quite a few child and adolescent psychiatry articles are published in journals for paediatrics and general psychiatry.

 Secondly Clinics of North America usually publish articles which are usually review of the literature about different issues.

I have read this submission. I believe that I have an appropriate level of expertise to confirm that it is of an acceptable scientific standard.

# Peer review: Referee response for version 1

**DOI:** 10.5256/f1000research.13058.r24874

**Published:** 2017-08-10

**Authors:** Miyuru Chandradasa, Layani Champika

**Affiliations:** 1 Department of Psychiatry, Faculty of Medicine, University of Kelaniya, Ragama, Sri Lanka; 2 Colombo North Teaching Hospital, Ragama, Sri Lanka; 3 Latrobe Regional Hospital, Traralgon, Australia

The publication, Child psychiatry: A scientometric analysis 1980-2016 was read with enthusiasm and admiration. It is true that child and adolescent psychiatry significantly lags behind general adult psychiatry in producing valid clinically useful research. Assessing research output in a scientific field is of utmost importance as it provides a guide for the future development of that field. This article demonstrates the research output trends in child and adolescent psychiatry over the previous four decades. It allows us to generate an understanding of the future research trends and the gaps that should be addressed by researchers in this field. 

 The mapping of research output in a scientific field is an extremely difficult task. This is made even more cumbersome in the field of child and adolescent psychiatry, as there is an overlap of articles in fields of child psychiatry, child psychology, neuro developmental psychiatry and paediatrics. The authors have employed a sound technique to achieve this complex task by using reputed software such as Web of Science core collection records, Network Analysis Interface for Literature Studies Project scripts and Citespace. 

 The article highlights the emergence of new concepts in the discipline of child and adolescent psychiatry over the decades. It is interesting to note that some older publications influencing the field after many years from their initial publication. 

 Few limitations were considered for this publication. The authors have analysed articles of the journals with high impact factor at present. However, it should be noted that in the past different journals had higher impact factors for child and adolescent psychiatry. Many decades back certain reputed journals in general psychiatry published significant numbers of articles in child and adolescent psychiatry as journals dedicated only for this sub speciality were less popular. It is understandable logically that the authors selected the seven journals according to the current impact factors to make this project practical. 

 In relevance to the discussion, it would have been interesting to compare some aspects of the results of this study with similar scientometric analyses in other medical disciplines and General Psychiatry. The authors could have concentrated on research output related to aetiology, symptomatology and management compared to other fields of psychiatry and medicine. In future, it would be exciting to compare these results with other sub specialities in psychiatry such as forensic psychiatry, mental health related to intellectual disability, addiction psychiatry and old age psychiatry.

 In conclusion, the authors have made a valid and practical attempt to assess and analyse the research output in the discipline of child and adolescent psychiatry over the past four decades. Further scientometric analyses are required to assess the impact of other influential publications in child and adolescent psychiatry published in journals known for general or other specified medical specialities.

We have read this submission. We believe that we have an appropriate level of expertise to confirm that it is of an acceptable scientific standard.
